# Metabolic Alterations in Spheroid-Cultured Hepatic Stellate Cells

**DOI:** 10.3390/ijms21103451

**Published:** 2020-05-13

**Authors:** Koichi Fujisawa, Taro Takami, Nanami Sasai, Toshihiko Matsumoto, Naoki Yamamoto, Isao Sakaida

**Affiliations:** 1 Department of Liver Regenerative Medicine, Yamaguchi University School of Medicine, Ube Yamaguchi 755-8505, Japan; fujisawa@yamaguchi-u.ac.jp; 2 Department of Gastroenterology and Hepatology, Yamaguchi University Graduate School of Medicine, Ube Yamaguchi 755-8505, Japan; nao-yama@yamaguchi-u.ac.jp (N.Y.); sakaida@yamaguchi-u.ac.jp (I.S.); 3 Department of Laboratory Science, Faculty of Laboratory Science, Yamaguchi University Graduate School of Medicine, Ube Yamaguchi 755-8505, Japan; b006up@yamaguchi-u.ac.jp; 4 Department of Oncology and Laboratory Medicine, Yamaguchi University Graduate School of Medicine, Ube 755-8505, Japan; tm0831@yamaguchi-u.ac.jp; 5 Yamaguchi University Health Administration Center, Yamaguchi 753-8511, Japan

**Keywords:** hepatic stellate cell, microgravity, transcriptome, spheroid, metabolome, αSMA, YAP

## Abstract

Hepatic stellate cells (HSCs) play a vital role in liver fibrosis, and a greater understanding of their regulation is required. Recent studies have focused on relationships between extracellular matrix (ECM) stiffness and gene expression or cellular metabolism, but none have provided a detailed metabolic analysis of HSC changes in spheroid cultures. Accordingly, in the present study, we created an HSC spheroid culture and analyzed changes in gene expression and metabolism. Expression of α-smooth muscle actin (α-SMA) decreased in the spheroids, suppressing proliferation. Gene expression analysis revealed the cell cycle, sirtuin signaling, mitochondrial dysfunction, and the Hippo pathway to be canonical pathways, believed to result from decreased proliferative ability or mitochondrial suppression. In the Hippo pathway, nuclear translocation of the yes-associated protein (YAP) was decreased in the spheroid, which was associated with the stiffness of the ECM. Metabolome analysis showed glucose metabolism changes in the spheroid, including glutathione pathway upregulation and increased lipid synthesis. Addition of the glycolytic product phosphoenolpyruvate (PEP) led to increased spheroid size, with increased expression of proteins such as α-SMA and S6 ribosomal protein (RPS6) phosphorylation, which was attributed to decreased suppression of translation. The results of our study contribute to the understanding of metabolic changes in HSCs and the progression of hepatic fibrosis.

## 1. Introduction

Among liver cells, hepatic stellate cells (HSCs) are intralobular connective tissue cells that express surface structures of myofibroblasts or lipocytes, playing roles in homeostasis of the liver’s extracellular matrix (ECM), repair, regeneration, retinol metabolism, storage, and excretion [1]. Following liver damage, HSCs transform into myofibroblasts and become the main source of collagen, which is necessary for liver fibrosis, thereby contributing to intrahepatic portal hypertension [2]. Proliferation and migration of HSCs, as well as chemokine expression, play a role in the pathogenesis of hepatic inflammation and fibrosis; an improved understanding of the regulation of HSC activation may lead to a decreased burden of chronic liver damage and decreased mortality rate.

Recent studies have focused on the relationship between the extracellular environment and metabolism, finding that mechanical signals promote oncogenesis and change cellular metabolism, and that in epithelial cells [3], placement in a stiff matrix upregulates the key glycolytic protein phosphofructokinase, thereby increasing glycolysis [4]. It is reported that the Hippo pathway and its effector yes-associated protein (YAP) are important for stellate cell activation [5]. Further, it is known that the activity level of yes-associated protein/transcriptional coactivator with PSD95, DLG1, ZO-1 (PDZ)-binding motif (YAP/TAZ) differs according to the stiffness of the ECM, and to the cellular morphology, and that stiffness of the ECM can cause YAP/TAZ to translocate to the nucleus and cause changes in the expression of various genes [6]. Cysteine-rich angiogenic inducer 61 (Cyr61) is a target gene product of the Hippo-YAP/TAZ transcription pathway, and Cyr61 is a member of CCN (Cysteine-rich 61(Cyr61), connective tissue growth factor (CTGF), nephroblastoma overexpressed (NOV)) family that contains 6 highly homologous extracellular matrix (ECM) proteins involved in cell adhesion signaling [7].

The spheroid, which is a configuration of 3D cell culture that exploits intercellular adhesion, yields results closer to those in vivo compared to monolayer cell culture, and has potential application in drug screening and evaluation of liver function [8]. Numerous methods exist to generate spheroid cell cultures, including the nonadhesive surface, hanging drop, spinner flask, rotary, and centrifugation pellet methods. Several studies have reported the use of spheroids to study hepatic cells, including cultures of hepatocytes only, as well as coculture studies with HSCs and Kupffer cells. One study reported the creation of a 3D hepatocyte/HSC coculture spheroid using low-adhesion poly-DL-lactide [9]. Another study reported that the coculture system suitably mimicked the in vivo environment, showing utility for the prediction of in vivo hepatotoxicity [10]. Furthermore, organoids derived from the coculture of hepatocytes and HSCs have facilitated the evaluation of liver fibrosis [11], and spheroids created from cocultures of induced pluripotent stem cell-derived HSCs and hepatocytes have similarly been useful for studying liver fibrosis [12]. However, while there are reports of cocultures with hepatocytes, only a few studies have examined changes specifically in HSCs in spheroid cultures. It has been demonstrated that mRNA expression of α-smooth muscle actin (α-SMA), connective tissue growth factor (CTGF), and ankyrin repeat domain 1 (ANKRD1) decreases [5], but no other detailed analyses have been reported. Furthermore, the study of metabolic changes in spheroid cultures has been limited to those such as glycolytic shifts in spheroid-cultured cancer cells [13], with no reports of detailed studies in HSCs. 

In this study, we developed a novel method to create HSC spheroids that is based on monolayer cell culture with the non-adhesive surface method [14], and analyzed gene expression and the metabolome, thereby evaluating changes in protein synthesis and metabolism. We believe that our findings contribute toward understanding the relationship between the extracellular environment and metabolism in HSCs, evaluation of drugs related to hepatic fibrosis, and efficient production of hepatic organoids.

## 2. Results

### 2.1. α-SMA Expression Decreases in Spheroid Culture

**Figure 1:**
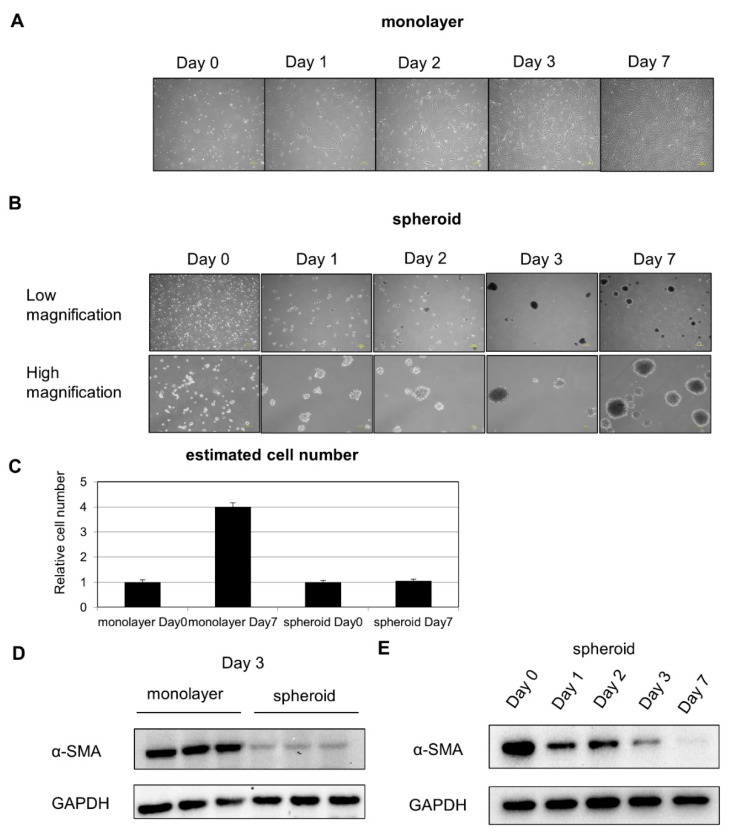
HSC composition over time in monolayer and spheroid cultures: (**A**) changes over time in HSCs in monolayer culture, 200 μm scale. (**B**) Changes over time in HSCs in spheroid culture. Upper panel: low power, 200 μm scale. Lower panel: high power, 100 μm scale. (**C**) Comparison of cell number on Day 0 and Day 7. (**D**) α-SMA expression in monolayer and spheroid cultures, Day 3. (**E**) Changes in α-SMA expression over time in the spheroid culture.

After placement in suspension culture system using a nonadhesive poly-hema-coated dish, floating HSCs began to aggregate gradually, leading to the formation of a spheroid (Figure 1A,B). Cell proliferation was inhibited in spheroid culture (Figure 1C). Protein was extracted from cells on Day 3 of the suspension culture, and Western blot analysis revealed a decrease in expression of the activated HSC marker α-SMA (Figure 1D). To evaluate changes over time, Western blot analysis was performed, and we found that α-SMA expression continued to decline with time (Figure 1E).

### 2.2. Gene Expression Analysis Results Demonstrate Changes in Metabolism

**Figure 2:**
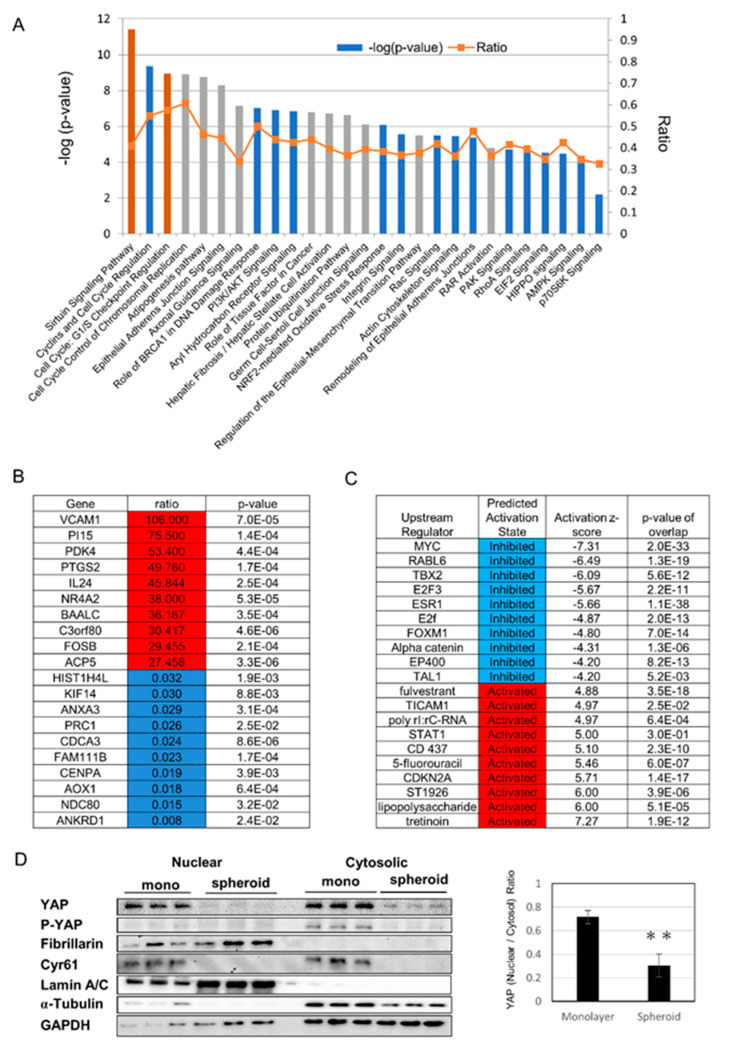
Gene expression analysis using serial analysis of gene expression (SAGE): (**A**) selected significantly enriched canonical pathways identified by ingenuity pathway analysis (IPA). The diagram shows significantly overrepresented canonical pathways. A multiple-testing corrected p-value was calculated using the Benjamini–Hochberg method to control the rate of false discoveries in statistical hypothesis testing. The ratio value represents the number of molecules in a given pathway that meet the cut-off criteria, divided by the total number of molecules that belong to the function. The brown bar indicates positive z-score, blue bar indicates negative z-score, and grey bar indicates no activity pattern available. (**B**) Top 10 and bottom 10 list of genes with major changes in expression, with increased expression shown in red and decreased expression shown in blue. (**C**) Among upstream regulators identified by IPA, the top five and bottom five expected activators are shown in red and expected inhibitors are shown in blue. (**D**) (Left) Protein expression by nuclear and cytosolic compartments. Cyr61 is a downstream gene of YAP. Lamin A/C is a nuclear marker and fibrillarin is a nucleolus marker. (Right) Ratio of nuclear YAP/Cytosolic YAP (yes-associated protein), ** *p* ≤ 0.01.

We chose Day 1 for RNA analysis to evaluate the early change of mRNA expression, because the protein expression of alpha-SMA decreased from Day1 (Figure 1E). Monolayer cells on Day 1 and spheroid cells on Day 1 were analyzed for gene expression using serial analysis of gene expression (SAGE), followed by ingenuity pathway analysis (IPA). Enriched canonical pathways included cell cycle pathways, the sirtuin pathway, eukaryotic translation initiation factor 2 (EIF2) signaling, mitochondrial dysfunction, epithelial adherence junction signaling, and Hippo signaling (Figure 2A). Genes with major changes in expression are shown in Figure 2B, and include the increased expression of the transmembrane protein, vascular cell adhesion molecule-1, and the glucose metabolism regulator, PDK4, as well as downregulation of ANKRD1, which is a downstream gene of YAP. Genes thought to be upstream regulators are shown in Figure 2C, and included increased activity of the vitamin A derivative all-*trans* retinoic acid (a form of retinoic acid in which all double bonds are in the *trans* configuration), as well as suppression of myelocytomatosis oncogene cellular homolog (MYC), which is thought to be involved in changes in mitochondrial metabolism. To evaluate the Hippo pathway, we used Western blotting to study nuclear translocation of YAP, and found that YAP and p-YAP, and nuclear YAP in particular, were decreased in the spheroid. Cyr61, which is downstream of YAP, also showed decreased expression in the spheroid (Figure 2D).

### 2.3. Glucose Metabolism Undergoes Changes with Upregulation of the Glutathione Pathway in Spheroid Culture

**Figure 3:**
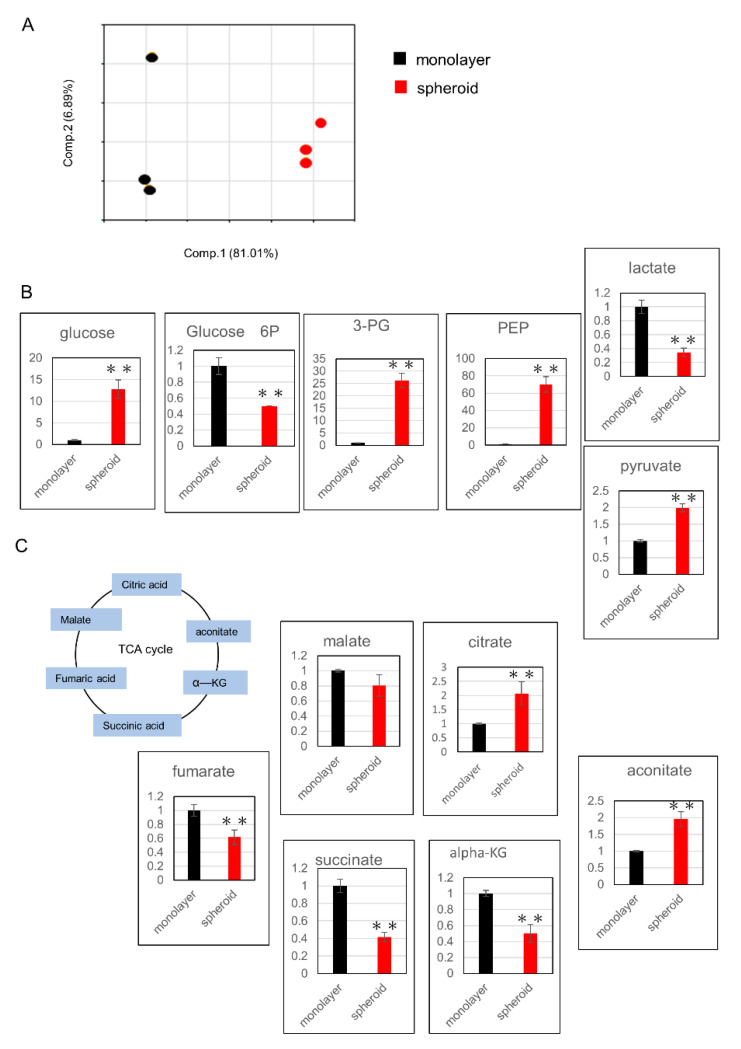
Assessment of metabolic changes using metabolome analysis: (**A**) principal component analysis of normalized metabolic data. Percentage values indicated on the axes represent the contribution rate of the first (PC1) and second (PC2) principal components to the total amount of variation. (**B**) Changes in metabolic products related to the glycolysis pathway. The y-axis represents relative intensity. (**C**) Changes in metabolic products related to the TCA cycle. The y-axis represents relative intensity. ** *p* ≤ 0.01. 3-PG: 3-phosphoglycerate, PEP: phosphoenolpyruvate, TCA: tricarboxylic acid, αKG: α-ketoglutarate.

Cells from Day 3 of the spheroid, as well as monolayer culture cells, were evaluated for metabolic products using metabolome analysis. All metabolites evaluated in this study were listed in Table S1. PCA analysis showed clear differences between the two groups (Figure 3A). Next, we examined changes in metabolic products within the glycolysis pathway and found that the levels of 3-phosphoglycerate (3-PG), phosphoenolpyruvate (PEP), and pyruvate were increased (Figure 3B). Among metabolic products of the tricarboxylic acid (TCA) cycle, citrate and aconitate levels were increased, while the levels α-ketoglutarate (αKG), succinate, fumarate, and malate were decreased (Figure 3C).

**Figure 4:**
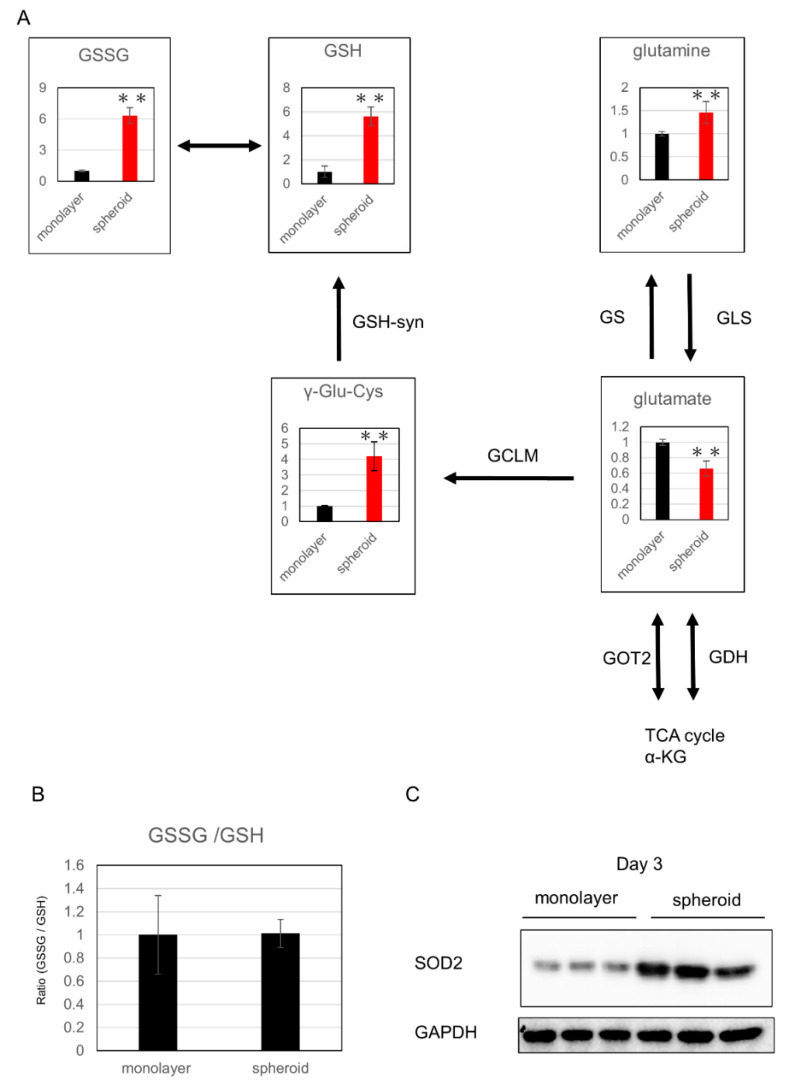
Evaluation of oxidative stress: (**A**) changes in metabolic products related to glutathione synthesis; the y-axis represents relative intensity. ** *p* ≤ 0.01. The enzymes that catalyze reactions were listed beside the arrows. αKG: α-ketoglutarate, GSH: glutathione-SH, GSSG: glutathione-S-S-Glutathione, γ-glu-cys: γ-glutamylcysteine, GS: glutamine synthetase, GLS: glutaminase, GOT: glutamic oxaloacetic transaminase, GDH: glutamate dehydrogenase, glutamate-cysteine lygase modified subunit. (**B**) GSSG/GSH ratio. (**C**) Western blot analysis of SOD2 expression.

Among the metabolic products of the glutathione synthesis pathway, which is related to oxidative stress, glutamine, γ-glutamylcysteine (γGluCys), reduced glutathione (GSH), and oxidized glutathione (GSSG) were significantly increased in the spheroid, whereas glutamic acid and αKG were significantly decreased, suggesting upregulation of glutathione metabolism in the spheroid (Figure 4A,B). Further, expression of superoxide dismutase 2 (SOD2), which is related to oxidative stress, was increased in the spheroid, which suggests a reaction to greater oxidative stress in the spheroid culture (Figure 4C).

### 2.4. Lipid Synthesis Is Upregulated in Spheroid Culture

**Figure 5:**
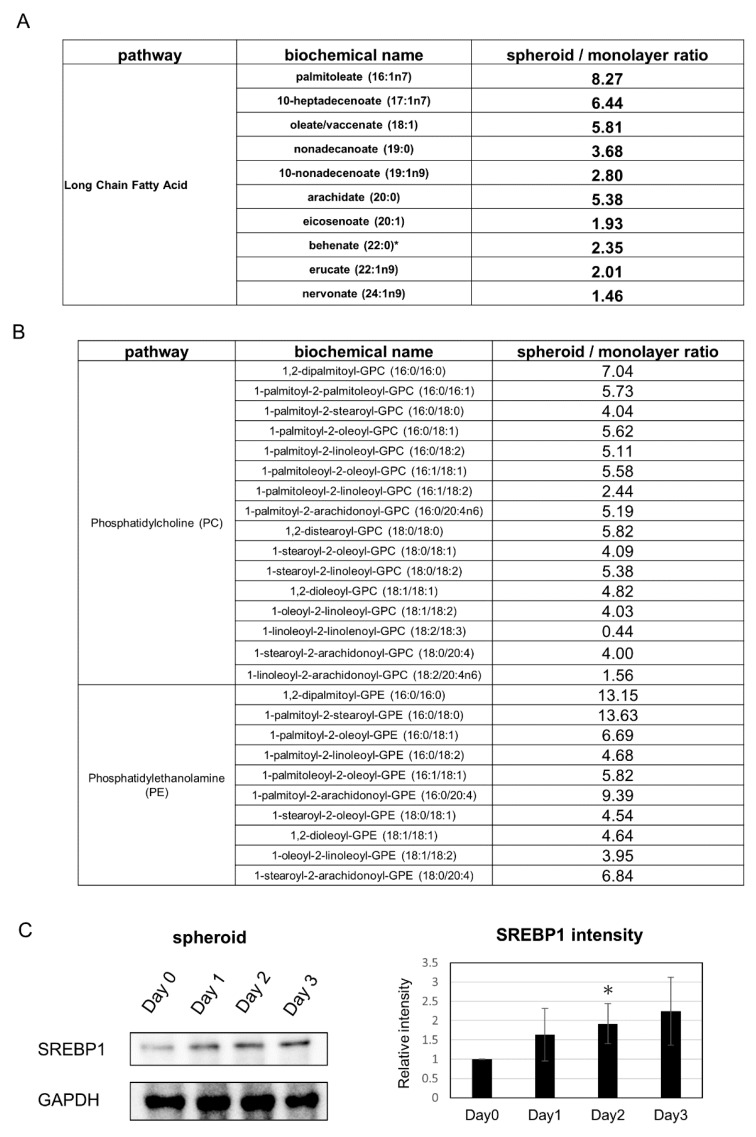
Changes in metabolic products related to lipid metabolism: (**A**) Changes in saturated long-chain fatty acids. (**B**) Changes in phosphatidylcholine (PC) and phosphatidylethanolamine (PE). (**C**) (Left) Western blot analysis of SREBP1 expression. (Right) Quantification of SREBP1 expression. * *p* ≤ 0.05, compared to Day 0.

Analysis of changes related to lipids showed that many saturated long-chain fatty acids were increased in the spheroid (Figure 5A). Further, cell membrane lipids such as phosphatidylcholine and phosphatidylethanolamine were also increased (Figure 5B). The expression of phosphocholine cytidylyltransferase, the rate-limiting enzymes in the cytidine 5’-diphosphate-choline pathway for phosphatidylcholine synthesis, tended to be upregulated in the SAGE analysis. In addition, SAGE analysis revealed that expression of the fatty acid synthesis related gene SREBP1, a transcriptional activator required for lipid homeostasis, was increased in the spheroid and adipogenesis pathway was observed in canonical pathways; subsequent Western blot analysis revealed an increased tendency of SREBP1 expression (Figure 5C). 

### 2.5. Addition of PEP Leads to Increased Spheroid Size

**Figure 6:**
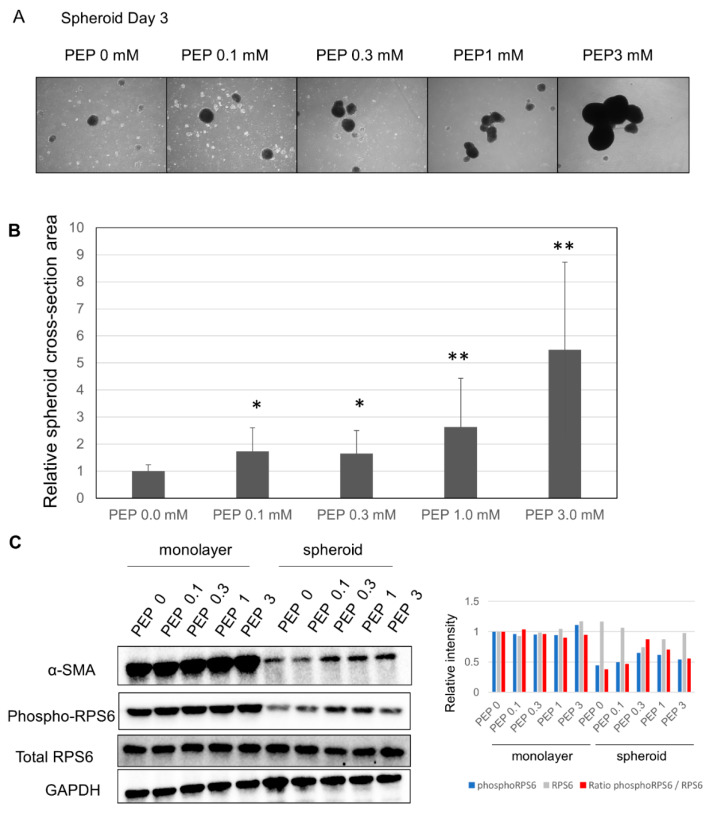
Changes in the spheroid due to the addition of PEP: (**A**) changes in the spheroid due to the addition of PEP; 200 μm scale. (**B**) Evaluation of spheroid surface area following the addition of PEP; * *p* ≤ 0.05, and ** *p* ≤ 0.01. (**C**) (Left) Changes in α-SMA, phospho-RPS6 (S6 ribosomal protein) and total RPS6 expression following the addition of PEP by Western blotting. (Right) Quantification of phosphoRPS6, total RPS6, and the ratio of phosphoRPS6/total RPS6.

After finding p70 S6 kinase pathway in canonical pathway with an inhibited z-score in the spheroid culture (Figure 2A), we considered whether suppression of the TCA cycle may have led to decreased ATP as well as downregulation of the pentose phosphate pathway (PPP), nicotinamide adenine dinucleotide phosphate reduced form (NADPH) synthesis, and amino acid synthesis. This led us to evaluate the effects of adding PEP, a high-energy metabolic product of the last step of glycolysis, which is able to pass through the cell membrane [15]. The size of the spheroid increased compared to the spheroid cultured without PEP (Figure 6A). Evaluation of size changes showed that, at 0.1 and 0.3 mM, the size of the spheroid grew by 1.7×; at 1.0 mM, the size grew by 2.6×; and at 3 mM, the size grew by 5.5× (Figure 6B). Further, Western blot analysis showed that α-SMA and phosphorylated S6 ribosomal protein expression were increased in the spheroid, suggesting upregulation of protein translation (Figure 6C).

## 3. Discussion

HSCs play a key role in liver fibrosis. When inactive, they are known to express epithelial markers and store vitamin A, whereas when active, these cells undergo epithelial-mesenchymal transition to myofibroblast-like cells. When cultured in vitro on polystyrene plates, HSCs become active, whereas culturing on soft support hinders activation, showing that the cellular microenvironment plays a key role in activation [16]. In the present study, we found that expression of the activated HSC marker α-SMA was greatly decreased with spheroid formation, suggesting suppression of activation.

It is thought that protein expression and metabolism differ greatly between monolayer and spheroid cultures, and it has been found that cancer cells in spheroid cultures display differential gene expression in low-oxygen environments, suggesting that oxygen concentration within the spheroid plays a role [17]. Here, gene expression analysis similarly showed that canonical pathways included the cell cycle, sirtuin pathway, and mitochondrial dysfunction, suggesting that proliferation was suppressed in the spheroid, with metabolic changes including mitochondrial suppression. Further, gene expression analysis showed increased expression of PDK4, an enzyme that regulates pyruvate dehydrogenase (PDH), which is responsible for the conversion of pyruvate to acetyl-CoA in glycolysis. PDK4 is thought to be a regulator controlling the balance between glycolysis and mitochondrial respiration, playing a role in suppression of mitochondrial activity. In cancer cells, detachment from the ECM leads to increased expression of PDK4, with PDH-mediated suppression of pyruvate metabolism [18]; thus, it is possible that a similar metabolic shift occurs in HSCs. Among upstream regulators, MYC level was observed to decrease, and MYC plays a role in upregulation of mitochondrial energy through protein synthesis as well as synthesis of mitochondria. Decreases in MYC expression have been reported to decrease cell death rates in low-oxygen or low-glucose states [19]. Furthermore, evaluation of TCA cycle metabolic products through metabolome analysis showed decreases in the levels of succinate, fumarate, and malate, with increases in those of citrate and aconitate. These same changes have been reported in TCA-cycle metabolome analysis in ovarian cancer cell spheroids [20]. In addition, it has been shown that de novo synthesis of fatty acids is upregulated simultaneously, and our study found that spheroid-cultured HSCs also showed accumulation of lipids with increased SREBP1, thus showing increased de novo synthesis of fatty acids. Oxidation of fatty acids leads to ATP generation, and fatty acids are also used to produce phospholipids, which compose the cell membrane. SAGE analysis showed that expression of a positive regulator of these processes, peroxisome proliferator-activated receptor γ, was significantly increased, as were the levels of the cell membrane constituents phosphatidylcholine and phosphatidylethanolamine. These data suggest increased de novo fatty acid synthesis in the spheroid for use in cell membrane maintenance.

In cancer cells, when nuclear factor erythroid 2-related factor 2 (NRF2) levels rise above those of MYC, antioxidant processes are activated in preparation for detachment [18]. Here, in the spheroid, Western blot analysis revealed increased SOD2 expression, and metabolome analysis revealed increased total levels of the glutathione metabolites GSH and GSSG. These results suggest that antioxidant changes occur in the HSC spheroid, possibly to counteract increased oxidative stress.

Activity levels of YAP/TAZ are related to the stiffness of the ECM and cell morphology; cells placed on stiff extracellular matrices show YAP/TAZ nuclear translocation that results in gene expression and subsequent cellular proliferation. In contrast, cells placed on soft extracellular matrices or shrunken cells with a low surface area exhibit cytosol-localized YAP/TAZ, with the absence of cellular proliferation [6]. YAP activity is known to be an important driver of HSC activation and is considered a possible target for suppression of hepatic fibrosis [18]. Our study also showed that spheroid cells exhibited decreased cytoplasmic and intranuclear YAP levels, as well as decreases in the levels of the downstream gene, Cyr61, showing increased activity of the Hippo pathway.

Reportedly, compared to monolayer cell culture, the interior of the spheroid constitutes a low-oxygen, low-glucose environment [21], leading to inefficient production of ATP at the center [22]. Accordingly, we hypothesized that increasing intracellular ATP may lead to the resumption of normal cell activity. However, as ATP cannot cross the cell membrane, one solution is the addition of PEP, the final, high-energy metabolic product of glycolysis, which is able to freely pass through the cell membrane [15]. We found that the addition of PEP led to increased spheroid size in a concentration-dependent manner. PEP has been shown to pass through red blood cell membranes and lead to increased ATP [23]; it is thought that addition of PEP to the spheroid may lead to increased ATP through conversion to pyruvate, as well as upregulation of the PPP, NADPH synthesis, and amino acid synthesis. Interestingly, inhibition of PCK, the enzyme responsible for PEP synthesis, leads to decreased spheroid size, suggesting that PEP is used for ribose synthesis, with synthesis of NADPH through the oxidative PPP [24].

The decrease in α-SMA expression in the spheroid is thought to be due to mechanical tension caused by increased stiffness of the ECM [25,26] or the inhibition of translation due to the effects of stretching forces on ER-associated protein translation pathways [27]. Our study showed markedly decreased α-SMA expression in the spheroid compared to the monolayer culture, which was thought to include inhibitory effects from both the stiffness of the ECM, as well as from the translational control. We think several pathways are related to α-SMA expression, and one significant pathway is translational control by ribosomal protein S6 kinase (p70S6K) [28]. It is reported that p-p70S6K expression was significantly correlated with α-SMA (a hallmark of myofibroblasts) expression in human pterygium fibroblasts (HPFs), while p70S6K knockdown reduced α-SMA protein levels in HPFs. They also suggested that p70S6K activation contributes to fibroblast transdifferentiation and maintains the myofibroblast phenotype in resident pterygium fibroblasts. Similar findings have been reported in liver fibroblasts [29]. Addition of PEP to the spheroid increased the levels of α-SMA, but not to the level of the monolayer culture; this was possibly due to the additional inhibitory effects of the stiffness of the ECM. However, further study is needed.

## 4. Materials and Methods

### 4.1. Cells and Cell Culturing

Human hepatic stellate cells (HHSteC) were purchased from ScienCell Research Laboratories, Inc., Carlsbad, CA, USA. Passage 3–7 cells were used for all experiments. Cells were maintained in DMEM medium (10% FBS) supplemented with 0.1 mM of Vitamin C. For spheroid cultures, cells were inoculated at a density of 5000 cells/cm^2^ on poly-hema-coated 10 cm plates. Poly-hema-coated plates were generated by the reported methods (Shri et al., 2018). Briefly, poly-hema (poly 2-hydroxyethyl methacrylate, P3932, Sigma, Tokyo, Japan) solution was made at a concentration of 12 mg/mL in 95% ethanol. The solution was kept at 37 °C overnight in a shaking incubator to dissolve the polymer completely. To cover the entire cell culture surface of the culture dish, the prepared poly-hema solution was layered. After drying, the wells were washed with sterile PBS two times and further sterilized by exposing to UV light for 30 min. Later, the culture plates were either used immediately or stored at 4 °C. Cell number was estimated with a DNA quantification kit (Cosmo Bio Co., Ltd. Tokyo, Japan).

### 4.2. Western Blot Analysis

Western blotting was conducted using standard methods. Briefly, cell lysis buffer was prepared with 62.5 mM Tris-HCl (pH 6.8), 4% sodium dodecyl sulfate (SDS), and 200 mM dithiothreitol; electrophoresis was performed using a 12% acrylamide gel. For electrophoresis, we used 20 μg of sample protein. After subsequently transferring the proteins to polyvinylidene fluoride (PVDF) membranes and applying a nonspecific epitope blocking using 5% skimmed milk, the following antibodies were applied for 1 h or overnight. Antibodies against α-SMA, YAP, p-YAP, Cyr61, α-tubulin, Lamin A, SOD2 and fibrillarin (Abcam, Tokyo, Japan); SREBP1, phosphoRPS6 and total RPS6 (CST, Tokyo, Japan); and glyceraldehyde 3-phosphate dehydrogenase (GAPDH) (Sigma-Aldrich, St. Louis, MO, USA) were used. Nuclear and cytosolic fractions were extracted using nuclear and cytoplasmic extraction regents (NE-PER) (Thermo Scientific Rockford, IL, USA).

### 4.3. Total RNA Isolation

RNA was collected and evaluated on Day 1. Total RNA was isolated from the cells using TRIzol reagent (Life Technologies, Carlsbad, CA, USA) and purified according to the manufacturer’s instructions. RNA samples were quantified using an ND-1000 spectrophotometer (NanoDrop Technologies, Wilmington, DE, USA) and the quality was confirmed using the Experion System (Bio-Rad, Hercules, CA, USA).

### 4.4. SAGE

The Ion AmpliSeq Transcriptome Human Gene Expression Kit (Life Technologies) was used to construct a library. An Ion PI IC 200 Kit (Life Technologies) and Ion PI Chip Kit v2 BC were used for sequencing, using an ion proton next-generation sequencer. The SAGE analysis was done once.

### 4.5. Metabolome Analysis

Metabolomic and statistical analyses were conducted at Metabolon, a commercial supplier of metabolic analysis, which has developed a platform that integrates chemical analysis (including identification and relative quantification), data reduction, and quality assurance. Cell pellets were subjected to methanol extraction, and split into aliquots for analysis. To maximize compound detection and accuracy, three separate analytical methods were utilized including ultra-high performance liquid chromatography-tandem mass spectrometry (UHPLC-LC-MS) in both positive and negative ion modes and gas chromatography/mass spectrometry (GC-MS) [30,31,32]. Metabolites were identified by automated comparison of ion features to a reference library of chemical standards, followed by visual inspection for quality control. The data were normalized by protein (Bradford). For statistical analyses and data display, any missing values were assumed to be below the limit of detection; these values were imputed with the compound minimum (minimum value imputation).

### 4.6. Statistical Analysis

Certain results were analyzed using Student’s t-test, and the data are presented as the mean ± standard deviation, with significance level established at *p* < 0.05. One-way ANOVA followed by Tukey’s post hoc test was used for statistical comparison between more than two groups. To determine statistical significance of metabolomic analysis, Welsh’s two-factor t-tests were performed in ArrayStudio (Omicsoft, Cary, NC, USA) or “R”, to compare protein-normalized data between experimental groups; *p* < 0.05 was considered significant.

## 5. Conclusions

In conclusion, we created a spheroid culture of HSCs and analyzed changes in gene expression and metabolism. Gene expression analysis showed a decreased proliferative ability in the spheroid, with metabolic changes, such as mitochondrial suppression and changes in the Hippo pathway that were related to the stiffness of the ECM. In addition, metabolome analysis showed changes in glucose metabolism in the spheroid, with upregulation of the glutathione pathway and lipid synthesis. Further, addition of the glycolytic metabolic product PEP led to increased spheroid size, with increased α-SMA expression, which was thought to be related to decreased inhibition of translation. Our results play an important role in understanding metabolic changes in HSCs and the role of these cells in hepatic fibrosis.

## Supplementary Materials

Supplementary materials can be found at https://www.mdpi.com/1422-0067/21/10/3451/s1. Table S1. The list of all metabolites evaluated in this study.

## Abbreviations

α-SMA	α-smooth muscle actin
ANKRD1	Ankyrin Repeat Domain 1
CYR61	cysteine-rich protein 61
HSC	Hepatic stellate cell
IPA	Ingenuity Pathway Analysis
PPP	pentose phosphate pathway
RPS6	S6 ribosomal protein
SAGE	serial analysis of gene expression
YAP/TAZ	Yes-associated protein/ transcriptional coactivator with PDZ-binding motif

## References

[1] Puche J.E., Saiman Y., Friedman S.L. (2013). Hepatic stellate cells and liver fibrosis. Compr. Physiol..

[2] Ezhilarasan D., Sokal E., Najimi M. (2018). Hepatic fibrosis: It is time to go with hepatic stellate cell-specific therapeutic targets. Hepatobiliary Pancreat. Dis. Int..

[3] Park J.S., Burckhardt C.J., Lazcano R., Solis L.M., Isogai T., Li L., Chen C.S., Gao B., Minna J.D., Bachoo R. (2020). Mechanical regulation of glycolysis via cytoskeleton architecture. Nature.

[4] Ayad N.M.E., Weaver V.M. (2020). Tension in tumour cells keeps metabolism high. Nature.

[5] Mannaerts I., Leite S.B., Verhulst S., Claerhout S., Eysackers N., Thoen L.F., Hoorens A., Reynaert H., Halder G., van Grunsven L.A. (2015). The Hippo pathway effector YAP controls mouse hepatic stellate cell activation. J. Hepatol..

[6] Dupont S. (2016). Role of YAP/TAZ in cell-matrix adhesion-mediated signalling and mechanotransduction. Exp. Cell Res..

[7] Schmeichel K.L., Bissell M.J. (2003). Modeling tissue-specific signaling and organ function in three dimensions. J. Cell Sci..

[8] Thomas R.J., Bennett A., Thomson B., Shakesheff K.M. (2006). Hepatic stellate cells on poly(DL-lactic acid) surfaces control the formation of 3D hepatocyte co-culture aggregates in vitro. Eur. Cells Mater..

[9] Choi Y.Y., Seok J.I., Kim D.S. (2019). Flow-Based Three-Dimensional Co-Culture Model for Long-Term Hepatotoxicity Prediction. Micromachines.

[10] Leite S.B., Roosens T., El Taghdouini A., Mannaerts I., Smout A.J., Najimi M., Sokal E., Noor F., Chesne C., van Grunsven L.A. (2016). Novel human hepatic organoid model enables testing of drug-induced liver fibrosis in vitro. Biomaterials.

[11] Coll M., Perea L., Boon R., Leite S.B., Vallverdu J., Mannaerts I., Smout A., El Taghdouini A., Blaya D., Rodrigo-Torres D. (2018). Generation of Hepatic Stellate Cells from Human Pluripotent Stem Cells Enables In Vitro Modeling of Liver Fibrosis. Cell Stem Cell.

[12] Rodriguez-Enriquez S., Gallardo-Perez J.C., Aviles-Salas A., Marin-Hernandez A., Carreno-Fuentes L., Maldonado-Lagunas V., Moreno-Sanchez R. (2008). Energy metabolism transition in multi-cellular human tumor spheroids. J. Cell. Physiol..

[13] Folkman J., Moscona A. (1978). Role of cell shape in growth control. Nature.

[14] Olsen A.L., Bloomer S.A., Chan E.P., Gaca M.D., Georges P.C., Sackey B., Uemura M., Janmey P.A., Wells R.G. (2011). Hepatic stellate cells require a stiff environment for myofibroblastic differentiation. Am. J. Physiol. Gastrointest. Liver Physiol..

[15] Kopp S., Sahana J., Islam T., Petersen A.G., Bauer J., Corydon T.J., Schulz H., Saar K., Huebner N., Slumstrup L. (2018). The role of NFkappaB in spheroid formation of human breast cancer cells cultured on the Random Positioning Machine. Sci. Rep..

[16] Buchheit C.L., Weigel K.J., Schafer Z.T. (2014). Cancer cell survival during detachment from the ECM: Multiple barriers to tumour progression. Nat. Rev. Cancer.

[17] Okuyama H., Endo H., Akashika T., Kato K., Inoue M. (2010). Downregulation of c-MYC protein levels contributes to cancer cell survival under dual deficiency of oxygen and glucose. Cancer Res..

[18] Liao J., Qian F., Tchabo N., Mhawech-Fauceglia P., Beck A., Qian Z., Wang X., Huss W.J., Lele S.B., Morrison C.D. (2014). Ovarian cancer spheroid cells with stem cell-like properties contribute to tumor generation, metastasis and chemotherapy resistance through hypoxia-resistant metabolism. PLoS ONE.

[19] Ishii A., Kimura T., Sadahiro H., Kawano H., Takubo K., Suzuki M., Ikeda E. (2016). Histological Characterization of the Tumorigenic "Peri-Necrotic Niche" Harboring Quiescent Stem-Like Tumor Cells in Glioblastoma. PLoS ONE.

[20] Walenta S., Dotsch J., Bourrat-Flock B., Mueller-Klieser W. (1990). Size-dependent oxygenation and energy status in multicellular tumor spheroids. Adv. Exp. Med. Biol..

[21] Golbidi S., Moriuchi H., Yang C., Irikura M., Irie T., Hamasaki N. (2003). Preventive effect of phosphoenolpyruvate on hypoxemia induced by oleic acid in Guinea pigs. Biol. Pharm. Bull..

[22] Yonenaga K., Todoroki H., Tokunaga K., Hamasaki N. (1986). Changes in adenosine triphosphate, 2,3 diphosphoglycerate, and P50 of dog blood following transfusion of autologous red cells pretreated with phosphoenolpyruvate in vitro. Transfusion.

[23] Leithner K., Hrzenjak A., Trotzmuller M., Moustafa T., Kofeler H.C., Wohlkoenig C., Stacher E., Lindenmann J., Harris A.L., Olschewski A. (2015). PCK2 activation mediates an adaptive response to glucose depletion in lung cancer. Oncogene.

[24] Arora P.D., Narani N., McCulloch C.A. (1999). The compliance of collagen gels regulates transforming growth factor-beta induction of alpha-smooth muscle actin in fibroblasts. Am. J. Pathol..

[25] Hinz B., Mastrangelo D., Iselin C.E., Chaponnier C., Gabbiani G. (2001). Mechanical tension controls granulation tissue contractile activity and myofibroblast differentiation. Am. J. Pathol..

[26] Sjuve R., Haase H., Ekblad E., Malmqvist U., Morano I., Arner A. (2001). Increased expression of non-muscle myosin heavy chain-B in connective tissue cells of hypertrophic rat urinary bladder. Cell Tissue Res..

[27] Zhao X.R., Zhang M.C., Xie H.T., Ji N., Sun L.T. (2018). p70S6K activation promotes the transdifferentiation of fibroblasts to myofibroblasts in pterygium tissue growth on the cornea. Biotechnol. Lett..

[28] Le Pabic H., L’Helgoualc’h A., Coutant A., Wewer U.M., Baffet G., Clement B., Theret N. (2005). Involvement of the serine/threonine p70S6 kinase in TGF-beta1-induced ADAM12 expression in cultured human hepatic stellate cells. J. Hepatol..

[29] Shin S.Y., Fauman E.B., Petersen A.K., Krumsiek J., Santos R., Huang J., Arnold M., Erte I., Forgetta V., Yang T.P. (2014). An atlas of genetic influences on human blood metabolites. Nat. Genet..

[30] Suhre K., Shin S.Y., Petersen A.K., Mohney R.P., Meredith D., Wägele B., Altmaier E., Deloukas P., Erdmann J., Grundberg E. (2011). Human metabolic individuality in biomedical and pharmaceutical research. Nature.

[31] Evans A.M., DeHaven C.D., Barrett T., Mitchell M., Milgram E. (2009). Integrated, nontargeted ultrahigh performance liquid chromatography/electrospray ionization tandem mass spectrometry platform for the identification and relative quantification of the small-molecule complement of biological systems. Anal. Chem..

[32] Ford L., Kennedy A.D., Goodman K.D., Pappan K.L., Evans A.M., Miller L.A., Wulff J.E., Wiggs B.R., Lennon J.J., Elsea S. (2020). Precision of a Clinical Metabolomics Profiling Platform for Use in the Identification of Inborn Errors of Metabolism. J. Appl. Lab. Med..

